# Influence of cluster thinning and girdling on aroma composition in ‘Jumeigui’ table grape

**DOI:** 10.1038/s41598-020-63826-7

**Published:** 2020-04-23

**Authors:** Xiaojun Xi, Qian Zha, Yani He, Yihua Tian, Aili Jiang

**Affiliations:** 1 Forestry and Pomology Research Institute, Shanghai Academy of Agricultural Sciences, Shanghai, 201403 China; 2 Shanghai Key Lab of Protected Horticultural Technology, Shanghai Academy of Agricultural Sciences, Shanghai, 201403 China

**Keywords:** Plant physiology, Secondary metabolism

## Abstract

Cluster thinning and girdling are common and simple practices applied to improve berry quality in table grape cultivation. However, there is limited information about the accumulation and biosynthesis of the entire aromatic profile under cluster thinning and girdling, notably in table grapes. This research investigated the influences of cluster thinning and girdling (alone or in combination) on aroma profiles, particularly the changes in biosynthesis and accumulation of Muscat-flavored related compounds from véraison to harvest in ‘Jumeigui’ grape. Cluster thinning and girdling (alone or in combination) significantly increased the concentrations of total soluble solids (TSS) and key aromatic compounds at harvest, with higher concentrations of both under cluster thinning than girdling. Berry weight and titratable acidity (TA) were unaffected by cluster thinning, girdling, or in combination at harvest. Linalool, the most abundant and active odorant related to Muscat flavor, accumulated in 28.6% and 20.2% higher concentrations from cluster thinning than control and girdling at maturity, respectively. Furthermore, higher *DXS3* transcript abundance in cluster thinning groups might contribute to the increased accumulation of terpenes and linalool in ‘Jumeigui’ grape. The results will contribute to further understand the mechanism of source/sink ratio modulation on aroma accumulation and better apply cluster thinning and girdling for grape production.

## Introduction

Aroma is one of the important indicators contributing to table grape and wine quality^1,2^. Although several hundred aroma compounds have been identified, the most important families of compounds responsible for the aroma of grapes are terpenes, C_13_ norisoprenoids, methoxypyrazines, C_6_ alcohols and aldehydes, esters, and thiols^1,3^. Aroma compounds in grapes exist as free and bound glycosides. In table grapes, free forms might be the only vital ingredients to determine the flavor despite odourless bound glycosylated forms could be hydrolyzed to odour-active free forms during fermentation^4,5^.

Monoterpene alcohols, notably linalool, with low perception thresholds, contribute to floral characters and are particularly prevalent in Muscat flavored varieties. Monoterpenes in grapevine berries have been demonstrated to biosynthesize via the plastidial 2-C-methyl-D-erythritol-4-phosphate (MEP) pathway^6^. Due to the correlation between gene expression and monoterpenes accumulation, 1-deoxy-D-xylulose 5-phosphate (DXP) synthase (DXS), DXP reductoisomerase (DXR), 4-hydroxy-3-methylbut-2-enyl diphosphate reductase (HDR) and geranyl pyrophosphate synthase (GPPS) are putative upstream rate-limiting enzymes^7–9^. Moreover, many of terpene synthases (TPSs) involved in late steps of monoterpenols biosynthesis have been functionally characterized^10,11^. Among them, the linalool/nerolidol synthase gene *VvCSLinNer* was reported to play an important role in linalool accumulation^8,12^.

The effects of agronomic practices such as leaf removal, irrigation, and exogenous hormones on primary and secondary metabolism in grapes have been well studied during the past decades^3^. Regulation of the source/sink ratio via cluster thinning is a common practice to enhance the accumulation of secondary metabolites, especially flavonols and anthocyanins^13–18^. However, there is little research concerning the impact of cluster thinning on aromatic compounds in berries. Cluster thinning enhanced sensory attributes in Grenache wines, but reduced them in Tempranillo wines^19^. The effect of cluster thinning on free and glycosylated volatile terpenes showed a significant increase in ‘Sauvignon Blanc’ berries, and the contents of terpenes were the highest in thinned treatment conducted one week before véraison^20^. Furthermore, girdling is another viticulture practice to improve grape quality through regulating the source/sink ratio^21–23^. Unfortunately, there is a lack of research about the influence of girdling on aroma profiles in grapes. Recently, peduncle girdling of post-veraison ‘Riesling’ bunches was reported to significantly increase monoterpenoid accumulation and reduce esters and higher alcohols^24^.

In China and other Asian countries, table grapes are mostly hybrid cultivars between *Vitis vinifera* and *Vitis labrusca*, owing to their high sugar levels and disease resistance^25^. Among them, ‘Jumeigui’, a progeny obtained from ‘Muscat Hamburg’ (4×) × ‘Kyoho’ (4×), is one of the most consumer-preferred table grape cultivars with rich Muscat flavor and planted widely in China. To obtain quality grapes with good appearance and desirable flavor, cluster thinning and girdling are common and efficient practices used in table grape cultivation. However, there is little information about the changes in aromatic profile accumulation and biosynthesis under cluster thinning and girdling, notably in table grapes. This work was aimed to explore and demonstrate the influences of cluster thinning and girdling (alone or in combination) on aroma profiles, as well as the changes in biosynthesis and accumulation of Muscat-flavored related compounds throughout the entire ripening period in ‘Jumeigui’ grape. The results will contribute to further understand the mechanism of source/sink ratio modulation on aroma accumulation and better apply cluster thinning and girdling for grape production.

## Results

### Yield, berry growth, and quality

Due to removal of half clusters, the yield per vine of thinned groups, CT (cluster thinning) and CTG (cluster thinning + girdling), were reduced by 48% and 49% against control group (CK), respectively (Table 1). There was no significant difference between G (only girdled group) and CK. In addition, the LA per vine was similar among the four treatments at harvest (Table 1). Likewise, the LA/Yield ratio was similar between G and CK. However, the LA/Yield ratio increased 0.9- and 1.0-fold in CT and CTG, respectively, compared to CK.

**Table 1:** Vine parameters under cluster thinning and girdling at harvest.

Treatment	Cluster/vine	Yield/vine (kg)	Leaf area/vine (m^2^)	Leaf area/yield (m^2^/kg)
CK	72a	37.52 ± 2.27a	18.84 ± 0.84a	0.50 ± 0.05a
G	72a	38.14 ± 1.79a	18.98 ± 0.65a	0.50 ± 0.04a
CT	36b	19.33 ± 1.09b	18.55 ± 1.31a	0.96 ± 0.12b
CTG	36b	19.00 ± 0.73b	19.06 ± 0.86a	1.00 ± 0.03b

CK, the control treatment (unthinned and ungirdled); G, trunk girdled one week before véraison; CT, 50% clusters thinned one week before véraison; CTG, 50% clusters thinned and trunk girdled one week before véraison. Different letters within a column indicate statistically different values (*p* < 0.05) according to Duncan’s test.

Berry weight was unaffected by cluster thinning or girdling during the ripening period (Table 2). Both cluster thinning and girdling increased TSS compared to CK, especially from 71 DAA to harvest. At full ripe, TSS in CTG, CT, and G were 19.7%, 12.7%, and 7.5% higher than CK, respectively. TSS of the thinned groups (CT) was higher than that of the girdled groups (G). TA in the thinned and girdled berries were similar with CK at the onset of véraison (57 DAA), except for the CTG with sharp dropping (Table 2). Then the TA of the thinned or girdled groups declined sharply at 64 DAA and there was no significant difference among G, CT and CTG from 64 DAA to harvest. The drop of TA in CK berries delayed and had no significant difference with other treatments until harvest.

**Table 2:** Berry fresh weight (FW, g), total soluble solid (TSS, °Brix) and titratable acidity (TA, g/L) of ‘Jumeigui’ grape under cluster thinning and girdling during berry ripening.

Physicochemical parameter	Treatment	Berry development days after anthesis (DAA)				
		57	64	71	78	85
FW	CK	6.3 ± 0.4a	6.8 ± 0.5a	7.1 ± 0.2a	7.7 ± 0.3a	7.7 ± 0.2a
	G	6.4 ± 0.5a	6.9 ± 0.1a	7.0 ± 0.2a	7.8 ± 0.2a	7.7 ± 0.1a
	CT	6.5 ± 0.3a	7.0 ± 0.4a	7.1 ± 0.3a	7.9 ± 0.3a	7.7 ± 0.2a
	CTG	6.7 ± 0.3a	7.0 ± 0.4a	7.1 ± 0.1a	8.1 ± 0.2a	7.9 ± 0.1a
TSS	CK	8.4 ± 0.5a	11.7 ± 0.2a	13.5 ± 0.1a	15.9 ± 0.1a	17.3 ± 0.1a
	G	8.9 ± 0.1ab	11.9 ± 0.1a	14.4 ± 0.2b	17.4 ± 0.1b	18.6 ± 0.2b
	CT	9.3 ± 0.5b	12.3 ± 0.5a	15.9 ± 0.1c	18.6 ± 0.2c	19.5 ± 0.2c
	CTG	10.2 ± 0.3c	13.6 ± 0.3b	16.3 ± 0.1d	19.9 ± 0.1d	20.7 ± 0.1d
TA	CK	17.1 ± 0.5b	11.2 ± 0.3b	9.2 ± 1.5b	6.7 ± 0.2b	5.8 ± 0.5a
	G	17.5 ± 0.2b	9.6 ± 0.3a	7.5 ± 0.2a	5.8 ± 0.4ab	5.7 ± 0.3a
	CT	17.0 ± 0.3b	8.8 ± 0.5a	7.6 ± 0.4a	5.4 ± 0.8a	5.6 ± 0.4a
	CTG	15.5 ± 0.9a	8.8 ± 1.2a	6.8 ± 0.1a	5.4 ± 0.4a	5.8 ± 0.8a

CK, the control treatment (unthinned and ungirdled); G, trunk girdled one week before véraison; CT, 50% clusters thinned one week before véraison; CTG, 50% clusters thinned and trunk girdled one week before véraison. Different letters within a column indicate statistically different values (*p* < 0.05) according to Duncan’s test.

### Aromatic compounds

Esters and C_6_ compounds were the predominant components of ‘Jumeigui’ grape at harvest, which accounted for 87.3–90.4% of the total components in the four treatments (Supplementary Fig. S1). However, the main component in berries at the onset of véraison were C_6_ compounds, which decreased gradually accompanied with increasing esters and terpenes from véraison to harvest. Likewise, the proportion of aldehydes declined constantly from the onset of véraison. In addition, the percentage of alcohols was negligible throughout the ripening period.

Regarding C_6_ compounds at maturity, the aroma-active compounds (OVAs > 1) were hexanal, (*Z*)-3-hexenal, (*E*)-2-hexenal and (*E*)-2-hexenol. Among them, hexanal was the most abundant and active compounds, which was accumulated at higher levels in G, CT and CTG against CK (Supplementary Table S1). Similarly, cluster thinning and girdling had positive effects on the contents of (*E*)-2-hexenal, (*Z*)-3-hexenal and (*E*)-2-hexenol in contrast with control. Despite the low concentration, (*Z*)-3-hexenal made positive contributions to the flavor of grape berries due to its low threshold (Supplementary Table S1). On the contrary, hexanol, detected at high levels, had nearly no contribution to the aroma owing to its high threshold. Furthermore, cluster thinning and girdling had significant impacts on the concentrations of total C_6_ compounds, which in CTG, CT and G were 48.9%, 36.4% and 13.3% higher than those in CK, respectively (Table 3).

**Table 3:** Concentrations (μg/kg) of volatile compounds under cluster thinning and girdling in the pulp juice of ‘Jumeigui’ grape at harvest.

Compounds	Treatment			
	CK	G	CT	CTG
**C**_**6**_ **compounds**				
Hexanal	1656 ± 48a	1860 ± 48b	2049 ± 30c	2084 ± 39c
(*Z*)-3-Hexenal	7.9 ± 2.0a	8.6 ± 2.3ab	10.9 ± 2.5ab	13.3 ± 3.1b
(*E*)-2-Hexenal	682 ± 23a	815 ± 31b	1095 ± 41c	1198 ± 50d
Hexanol	441 ± 30a	405 ± 45a	643 ± 61b	828 ± 40c
(*Z*)-3-Hexenol	11.9 ± 0.2a	16.2 ± 0.8b	15.0 ± 0.7b	14.8 ± 0.9b
(*E*)-2-Hexenol	134 ± 12a	217 ± 54b	189 ± 16ab	229 ± 53b
Subtotal	2933 ± 59a	3322 ± 91b	4001 ± 68c	4367 ± 92d
**Alcohols**				
1-Octen-3-ol	3.28 ± 0.16a	3.14 ± 0.12a	4.84 ± 0.29b	7.29 ± 0.32c
Heptanol	3.58 ± 0.26b	3.04 ± 0.11a	2.87 ± 0.19a	4.08 ± 0.31c
2-Ethyl hexanol*	1.63 ± 0.05a	1.37 ± 0.18a	1.27 ± 0.36a	1.60 ± 0.10a
Octanol	0.83 ± 0.11b	0.57 ± 0.07a	1.10 ± 0.11c	1.52 ± 0.18d
Phenylethyl alcohol	0.83 ± 0.24a	1.20 ± 0.13b	1.70 ± 0.02c	1.53 ± 0.11c
Subtotal	10.2 ± 0.4a	9.3 ± 0.5a	11.8 ± 0.2b	16.0 ± 0.8c
**Esters**				
Ethyl acetate	2275 ± 197a	3343 ± 88b	5159 ± 113d	4885 ± 134c
Ethyl propionate*	3.47 ± 0.23a	4.85 ± 0.21b	5.14 ± 0.64b	8.64 ± 0.98c
Ethyl isobutyrate	nd	15.1 ± 8.7a	27.2 ± 6.8a	55. 9 ± 7.0b
Ethyl butyrate	79.2 ± 10.5a	256 ± 6b	318 ± 21c	376. ± 32d
Ethyl 2-methylbutanoate*	2.26 ± 0.42a	8.95 ± 0.44b	12.2 ± 4.9b	13.1 ± 4.6b
Ethyl pentanoate	31.4 ± 3.3a	110 ± 3c	76.1 ± 10.1b	33.7 ± 4.5a
Ethyl hexanoate	29.7 ± 5.0a	62.9 ± 5.7bc	68.1 ± 7.0c	56.5 ± 4.4b
Hexyl acetate	1.18 ± 0.11a	1.96 ± 0.84ab	2.32 ± 0.35ab	3.31 ± 1.09b
2-Hexenoic acid ethyl ester*	3.24 ± 0.29a	5.55 ± 0.51b	11.0 ± 1.4c	14.6 ± 0.5d
Ethyl octanoate*	2.93 ± 2.10a	4.60 ± 0.16ab	6.49 ± 1.77b	6.02 ± 1.56ab
Ethyl 3-hydroxybutyrate*	1.84 ± 0.95a	4.49 ± 0.29b	6.11 ± 0.66c	9.23 ± 0.95d
Subtotal	2430 ± 212a	3817 ± 88b	5691 ± 126c	5462 ± 172c
**Aldehydes**				
Pentanal	4.96 ± 0.94ab	4.46 ± 0.61a	6.17 ± 0.46c	6.02 ± 0.83bc
Heptanal*	12.7 ± 3.6a	13.0 ± 2.3a	13.6 ± 2.8a	13.5 ± 1.7a
Octanal	6.82 ± 0.52a	6.76 ± 1.01a	8.90 ± 0.86b	8.13 ± 0.81ab
Nonanal	59.7 ± 15.1a	48.4 ± 3.2a	64.7 ± 8.1a	53.5 ± 7.1a
Benzaldehyde	17.3 ± 3.1a	16.3 ± 2.6a	17.8 ± 4.4a	17.0 ± 6.5a
Phenylacetaldehyde*	4.68 ± 0.21b	2.77 ± 0.62a	3.24 ± 0.25a	3.47 ± 0.17a
Subtotal	106 ± 17a	91.7 ± 10.0a	114 ± 16a	102 ± 10a
**Terpenes**				
Subtotal	659 ± 11a	722 ± 24b	906 ± 12c	999 ± 13d
Total	6138 ± 298a	7962 ± 149b	10725 ± 192c	10945 ± 231d

CK, the control treatment (unthinned and ungirdled); G, trunk girdled one week before véraison; CT, 50% clusters thinned one week before véraison; CTG, 50% clusters thinned and trunk girdled one week before véraison. Different letters within rows indicate statistically different values (*p* < 0.05) according to Duncan’s test. *Indicated the semi-quantitative determinations with the internal standards.

The total ester content in CTG, CT and G increased 1.2, 1.3 and 0.6-fold, respectively, compared to CK (Table 3). Four compounds with low threshold were the main contributors to flavor. Among them, ethyl butyrate was the most abundant significantly accumulated in G, CT and CTG berries compared with CK. Likewise, the concentrations of other three compounds, ethyl 2-methylbutanoate, ethyl pentanoate and ethyl hexanoate, were positively increased in cluster thinning and girdling groups. Interestingly, ethyl isobutyrate, was not detected in berries of CK, but contributed to aroma of berries in other three treatments, especially in CTG. Ethyl acetate was the most abundant ester, ranging from 87.6–93.6% of the total esters (Table 3). However, it had almost no contribution to aroma due to its high threshold (Supplementary Table S1).

The concentrations of each alcohol were at low intensity and only one compound, 1-octen-3-ol, was aroma-active but had little contribution to flavor. Cluster thinning significantly enhanced the concentration of 1-octen-3-ol, while girdling did not in contrast with CK (Table 3). Although both of proportion and content of aldehydes compounds also present at low level, there were three aroma-active compounds, especially nonanal, which made significant contributions to aroma owing to the low thresholds (Supplementary Table S1). However, cluster thinning and girdling had no significant impact on the concentrations of these compounds (Table 3).

Due to the pleasant Muscat-flavor of ‘Jumeigui’ grape, the impacts of cluster thinning and girdling (alone or in combination) on aromatic compounds were focused on terpenes throughout the ripening period (Table 4). Linalool was the most abundant and active odorant among the detected terpenes compounds. In contrast to other terpenes compounds, linalool dramatically accumulated from the onset of véraison and then accounted for 60.1–66.3% of total terpenes in the four treatments at harvest. Girdling did not significantly increase the concentration of linalool at the beginning of véraison until 78 DAA, whereas cluster thinning did throughout the ripening period (Table 4). At harvest, the berries of CTG, CT and G accumulated 37.4%, 28.6% and 7.1% linalool more than that of CK, respectively. In addition, the contents of linalool in thinned groups, especially in CTG, were significantly higher than that in girdled groups from véraison to harvest. The other aroma-active compounds, including D-limonene, cis-linalool oxide, and geraniol, were also significantly accumulated in thinned berries throughout the ripening period. Although girdling could promote the concentrations of these compounds against CK, there were no significant differences between G and CK at most developmental stages (Table 4). Interestingly, β-myrcene had no contributions to aroma in G and CK berries, whereas did positive contributions in CT and CTG. Furthermore, the berries of G, CT and CTG accumulated 9.5%, 37.5% and 51.6% terpenes more than that of CK at harvest, respectively (Table 3).

**Table 4:** Concentrations (μg/kg) of terpenes compounds under cluster thinning and girdling in the pulp juice of ‘Jumeigui’ grape during berry development.

Compounds	DAA	Treatment			
		CK	G	CT	CTG
β-Myrcene	57	11.8 ± 3.5a	12.0 ± 3.1a	13.9 ± 3.3ab	18.3 ± 0.9b
	64	19.4 ± 3.5a	15.3 ± 1.3a	16.9 ± 0.9a	17.5 ± 3.2a
	71	13.1 ± 1.5a	18.0 ± 1.7a	17.7 ± 0.8a	25.5 ± 5.0b
	78	24.1 ± 1.1a	25.1 ± 0.9a	38.4 ± 1.8b	34.5 ± 3.7b
	85	22.6 ± 3.6a	24.8 ± 1.2a	51.6 ± 6.0b	50.2 ± 8.4b
D-Limonene	57	54.3 ± 0.8a	56.5 ± 12.1a	54.3 ± 11.1a	68.6 ± 3.5a
	64	74.3 ± 4.4a	84.9 ± 4.1b	90.8 ± 1.1bc	95.4 ± 5.9c
	71	67.2 ± 4.8a	83.9 ± 10.0b	90.5 ± 8.0b	97.1 ± 7.7b
	78	73.6 ± 11.0a	82.6 ± 10.5a	86.4 ± 4.1a	88.4 ± 5.5a
	85	71.1 ± 4.3a	79.5 ± 6.3a	91.9 ± 4.6b	96.6 ± 7.0b
β-cis-Ocimene*	57	6.25 ± 0.53a	6.03 ± 0.38a	7.56 ± 0.68a	7.11 ± 1.48a
	64	12.0 ± 1.9ab	10.2 ± 1.2a	13.7 ± 1.8b	15.0 ± 1.5b
	71	11.5 ± 3.3a	12.3 ± 2.6a	15.9 ± 4.0a	14.9 ± 0.8a
	78	11.6 ± 2.7a	12.7 ± 2.8ab	17.2 ± 2.8bc	17.5 ± 0.9c
	85	12.2 ± 1.2a	13.6 ± 0.4ab	15.5 ± 1.2b	16.2 ± 2.6b
p-Cymene	57	nd	nd	nd	nd
	64	nd	nd	1.72 ± 0.06a	2.52 ± 0.41b
	71	2.27 ± 0.10a	3.28 ± 0.32b	3.17 ± 0.46b	3.10 ± 0.25b
	78	1.52 ± 0.28a	2.71 ± 0.22b	3.16 ± 0.11b	2.75 ± 0.74b
	85	1.87 ± 0.39a	2.69 ± 0.56ab	3.32 ± 0.40b	3.44 ± 0.38b
cis-Linalool oxide*	57	nd	nd	nd	nd
	64	nd	nd	nd	nd
	71	nd	nd	nd	nd
	78	nd	nd	2.00 ± 0.22a	2.28 ± 0.01b
	85	1.77 ± 0.07b	1.47 ± 0.05a	2.75 ± 0.17c	2.96 ± 0.09d
Linalool	57	1.77 ± 0.09a	2.40 ± 0.05b	5.08 ± 0.16c	5.96 ± 0.17d
	64	16.4 ± 2.5b	13.9 ± 0.2a	51.9 ± 0.6c	58.8 ± 0.6d
	71	74.7 ± 2.3a	72.7 ± 6.7a	155 ± 6b	170 ± 1c
	78	198 ± 3a	220 ± 5b	301 ± 6c	397 ± 4d
	85	437 ± 6a	468 ± 5b	562 ± 8c	600 ± 8d
α-Terpineol	57	1.79 ± 0.34a	2.05 ± 0.29a	2.15 ± 0.24a	2.11 ± 0.25a
	64	2.65 ± 0.55a	2.43 ± 0.09a	2.67 ± 0.05a	2.58 ± 0.13a
	71	5.98 ± 0.37a	5.59 ± 0.48a	12.3 ± 0.1b	14.3 ± 0.4c
	78	7.49 ± 0.06a	8.43 ± 0.23a	17.7 ± 2.2b	16.3 ± 3.4b
	85	16.4 ± 0.5a	18.2 ± 3.4ab	19.5 ± 0.9ab	20.5 ± 0.5b
Nerol	57	nd	nd	nd	nd
	64	nd	nd	nd	nd
	71	nd	nd	12. 8 ± 0.3a	14.8 ± 0.7b
	78	9.19 ± 0.24a	10.0 ± 0.2a	22.3 ± 4.0b	22.6 ± 3.0b
	85	15.6 ± 0.4a	17.4 ± 0.6a	22.3 ± 1.0b	22.2 ± 1.7b
Geraniol	57	nd	nd	nd	nd
	64	nd	nd	30.2 ± 2.1a	31.1 ± 1.7a
	71	54.8 ± 8.2a	50.2 ± 7.3a	81.5 ± 1.1b	97.7 ± 4.1c
	78	62.8 ± 12.0a	68.7 ± 4.4a	107 ± 11b	118 ± 21b
	85	80.9 ± 5.9a	96.6 ± 11.5a	138 ± 2b	187 ± 17c

CK, the control treatment (unthinned and ungirdled); G, trunk girdled one week before véraison; CT, 50% clusters thinned one week before véraison; CTG, 50% clusters thinned and trunk girdled one week before véraison. Different letters within rows indicate statistically different values (*p* < 0.05) according to Duncan’s test. nd: not detected. * Indicated the semi-quantitative determinations with the internal standards.

For evaluating the changes in global aroma profiles under cluster thinning and girdling (alone or in combination), the OAVs of the aroma compounds with similar descriptors were grouped into aromatic series according to a previous report^2^. The aroma of ‘Jumeigui’ grapes was mainly characterized by herbaceous, fruity, floral, and sweet (Supplementary Fig. S2). The berries of G, CT and CTG enhanced 18.5%, 31.0% and 27.9% herbaceous series more than that of CK at harvest, respectively. The values of fruity series in G, CT, and CTG were 1.0-, 1.5-, and 1.6-fold higher than those in CK, respectively. In addition, cluster thinning increased 30.7% floral series and 28.6% sweet series compared to CK, respectively. However, the values of floral series and sweet series in berries were similar between G and CK.

### Transcript abundance of monoterpene biosynthesis-related genes

**Figure 1:**
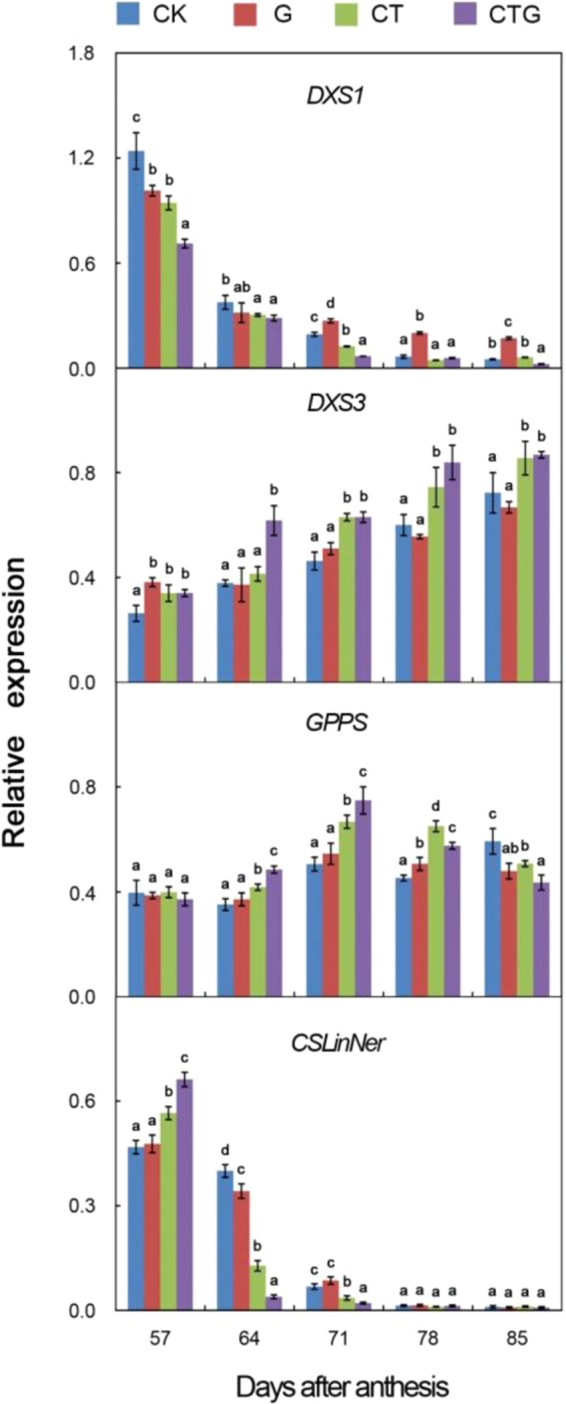
Expression levels of genes involved in monoterpene biosynthesis. CK, the control treatment (unthinned and ungirdled); G, trunk girdled one week before véraison; CT, 50% clusters thinned one week before véraison; CTG, 50% clusters thinned and trunk girdled one week before véraison. Data are mean ± SE of three biological replicates. Different letters on bar chart denotes statistical significance values (*p* < 0.05) according to Duncan’s test.

Among 6 genes involved in monoterpene biosynthesis, the expression pattern of *VvDXS1* and *VvCSLinNer* was similar with sharp drop of transcript levels from 64 DAA (Fig. 1). Till harvest, the expression of these two genes remained at low levels and was not correlated to total terpenes and linalool concentration. Contrarily, the transcript abundance of *VvDXS3* was significantly correlated with terpenes and linalool accumulation in all the four treatments (Supplementary Table S2). The expression of *VvDXS3* was significantly upregulated in berries of CT and CTG compared with CK, especially from 71 DAA. There was no significant difference between CK and G throughout the ripening period, except the beginning of véraison (57 DAA). Similarly, cluster thinning positively enhanced the transcript abundance of *VvGPPS* against CK from 64 to 78 DAA. Meanwhile, girdling only significantly upregulated its expression level at 71 DAA in contrast to CK. Interestingly, the transcript levels in CK berries were significantly higher than that in other treatments at harvest. Unfortunately, the transcript abundance of *VvDXR* and *VvHDR* was negligible in berries of all treatments throughout the ripening period.

## Discussion

Cluster thinning is a common viticulture technique of modulating the source-sink balance to improve berry quality and elevate the level of secondary metabolites^3^. Girdling is another viticulture technique involved in the source/sink ratio regulation, which directs the flow of phloem-transported metabolites to the fruit during the ripening stages. In this study, and in previous studies, girdling at the beginning of véraison advances fruit ripening and increase soluble solids with no yield reduction^22,26,27^. Likewise, cluster thinning significantly increased TSS of ‘Jumeigui’ berries consistent with previous studies^16,28–30^. Interestingly, in this study, TSS in thinned groups was significantly greater than that in girdled groups. An LA/Yield above 0.8 m^2^/kg was determined to be critical for full maturity of grape berries and the impacts of source/sink regulation on sugar accumulation^31^. In present study, cluster thinning boosted the LA/Yield ratio to 0.96 m^2^/kg, while girdling had an LA/Yield ratio of 0.50 m^2^/kg, similar to the control. Therefore, an LA/Yield ratio below 0.8 m^2^/kg in girdled groups might lower TSS compared to cluster thinning.

In this study, berry weight was the same across treatments throughout the ripening stages. Previous reports suggested cluster thinning and girdling had either no effect on berry weight^22,26,30^ or berry weight slightly increased^13,21,23^. The most of berry weight increasing achieved before applying treatment might contribute to similar berry weight across treatments in present study. Likewise, there were no significant differences in titratable acidity among the four treatments at harvest in the present study, similar to prior studies^22,30,32^. However, others have reported a slight increase^23,29^ or a significant decrease^26^ of TA in response to thinning and girdling. Some reports suggest that titratable acidity is less responsive to low LA/Yield ratios than sugars^30,33,34^. Therefore, both berry weight and titratable acidity might be insensitive to regulation of the source-sink balance with cluster thinning and girdling.

Despite a number of studies about the impact of cluster thinning on phenolic compounds in grapes, there is little research concerning effects on aroma composition, especially in table grapes. Cluster thinning was shown to be favourable for improvement of varietal aromas, including terpenes, ethyl esters, C_13_-norisoprenoids, and alcohols^20,35–37^. In this research on ‘Jumeigui’ grapes, cluster thinning significantly enhanced the concentrations of three primary components (C_6_ compounds, esters and terpenes) at harvest, consistent with prior results^20,35–37^. Grouping the OAVs of the aroma compounds with similar descriptors into aromatic series was reported to simplify the evaluation of global aroma profiles^2^. According to the OVAs and odorant series of 37 detected volatile compounds, the herbaceous, fruity, floral, and sweet series made up the aroma of ‘Jumeigui’ grape berries in present study. The values of those four odorant series were significantly increased under cluster thinning. Consequently, cluster thinning had a positive impact on flavor enhancement in ‘Jumeigui’ grape. However, recent research revealed that cluster thinning applied at the onset of véraison did not show a positive effect on grape sensory due to the limited effects on most aromatic compounds^38^. The high LA/Yield ratio in both of cluster thinning and control treatments was suggested to lead to similar berry maturation between the two treatments, which might contribute to limited differences in the concentrations of aroma and flavonol composition^17,38^. On the contrary, the LA/Yield ratio under cluster thinning was significantly greater than that under control in this study, probably leading to significantly increasing aroma compounds in thinned berries. In present research, girdling positively increased the values of herbaceous and fruity series, but had little impact on floral and sweet series. Interestingly, the values of those odorant series in thinned groups were greatly higher than the girdled groups. Unfortunately, to our knowledge, there is almost no information about the effect of girdling on aromatic compounds and the difference between cluster thinning and girdling. Therefore, the LA/Yield above 0.8 m^2^/kg was inferred to be crucial for comparing the impact of source-sink modulations on aroma accumulation as well as sugar. Previous reports have demonstrated that the concentrations of varietal compounds, especially monoterpenes, increased with positive correlation to soluble solids^35,39^. Consequently, the more aroma accumulation in thinned berries against girdled berries might be the consequence of higher TSS under cluster thinning with the LA/Yield above 0.8 m^2^/kg in this study.

In China, the ‘Jumeigui’ grape is widely planted and consumer-preferred due to its rich Muscat flavor, which are primarily attributed to extensive terpenes. Consistent with a previous report^2^, monoterpenes were detected in ‘Jumeigui’ grape with linalool as the most active odorant in pulp juice in this study. Moreover, cluster thinning significantly enhanced the contents of most terpenes, especially from 71 DAA, while girdling slightly increased in contrast with control. Like other compounds in this work, the concentration of terpene compounds under girdling was lower than that under cluster thinning. Previous reports have demonstrated that monoterpenes accumulation in grapevine berries were correlated with gene expression involved in MEP pathway, such as *DXS*, *DXR*, *HDR*, and *GPPS*^8,9,40,41^. However, in this study, only the expression level of *VvDXS3* showed significant correlation with terpenes and linalool accumulation in all the four treatments from véraison to harvest. *VvDXS3* has been suggested as a biomarker for monoterpene accumulation due to the high correlation coefficient between the monoterpene content and gene transcript abundance^40^. Consequently, the significant increase in *VvDXS3* transcript abundance under cluster thinning, especially from 71 DAA to harvest, might contribute to increased terpene and linalool accumulation against control, while no significant difference of gene expression between girdling and control at most ripening period might lead to the slight enhancement of terpenes and linalool levels under girdling. Interestingly, *VvDXS1* co-localized with a major QTL for monoterpene content^42^, only expressed at relatively high level at the onset of véraison and its transcript abundance was irrelevant to the increase of terpenes and linalool according to Pearson’s correlation coefficient analysis. No significant increase in *VvDXS1* transcript abundance related with monoterpene accumulation throughout the ripening period was also reported in ‘Gewürztraminer’ grapes^8^. Inconsistent with previous reports, *VvDXR* and *VvHDR* expression were not detected throughout the ripening stages in this study. Similarly, the transcript abundance of *VvCSLinNer* gene, which was reported as the key gene involved in linalool accumulation^8,12^, was very low throughout the ripening period except the onset of véraison, but was uncorrelated with linalool accumulation. The similar low expression levels of *VvCSLinNer* gene were found in ripening berries of other cultivars^11,43,44^. Therefore, these inconsistent reports on the same gene might due to the different cultivars and climatic conditions.

## Methods

### Plant materials and field trails

The experiments were performed during 2017 in an experimental vineyard at Shanghai Academy of Agricultural Sciences, Shanghai, China (30°51′N, 121°13′E) with table grape cultivar ‘Jumeigui’. The own-rooted grapevines were planted in the spring of 2008 with a north–south orientation and removed one by one at 4 m × 2.8 m spacing five years later. The vines were grown in a rain shelter with a Y-shape training system and managed according to the standard viticulture and disease control practices in Shanghai. The temperature (°C) and relative humidity (%) within the rain-shelter cultivation greenhouse were monitored hourly with data-loggers (HOBO U23-001, Onset Computer Inc., Bourne, MA, USA; Supplementary Fig. S3).

Thirty-six uniform vines were randomly selected and grouped into four treatments: control (CK), girdling (G), cluster thinning (CT), and cluster thinning + girdling (CTG).

The experimental design was a randomized block design with three vines as an experimental unit and replicated three times. Each vine retained 36 shoots and two clusters per shoot after bloom. In addition, each shoot and cluster was adjusted to 20 leaves and 60 berries at the pea-size stage, respectively. At 50 DAA (one week before véraison), 50% clusters of each vine in CT and CTG were removed with 36 clusters per vine left. Meanwhile, the single trunk of each vine in G and CTG was girdled by removing a 5 mm wide section of the phloem using a girdling knife from approximately 50 cm above ground level.

Berries were randomly sampled weekly from the onset of véraison which was one week after treatment (57 DAA, berries softening with ~8 °Brix) to harvest (85 DAA). For each biological replicate, 40 berries were randomly collected at each sampling date and half berries were randomly chosen for physicochemical analysis. The remaining berries were immediately frozen in liquid nitrogen and stored at −80 °C for aroma compound analysis and RNA extraction.

### Measurement of physicochemical parameters

Twenty berries of each biological replicate were sampled to determine berry fresh weight (FW; g), total soluble solids (TSS; °Brix) and titratable acidity (TA; g/L tartaric acid). TSS was estimated with a digital refractometer (PAL-1, Atago, Tokyo, Japan) and TA was measured through conventional acid-base titration.

### Measurement of vine performance

Fifteen clusters were randomly sampled from each replicate at harvest and weighed to estimate yield data. Five shoots per replicate were randomly selected after harvest and leaf area (LA) was measured with an LA meter (Yaxin-1241, Yaxinliyi Science and Technology, Beijing, China) to estimate average leaf area per shoot.

### Aroma compounds extraction and SPME-GC-MS analysis

Extraction and analysis of aromatic compounds was performed in Shanghai Jiao Tong University according to previously reported methods^2^. Briefly, deseeded pulps of ten berries were homogenized after thawing at 4 °C, centrifuged, and then filtered to achieve a clear juice. The extraction of aroma compounds in the juice was performed through headspace solid phase microextraction (HS-SPME) using a MPS-2 autosampler (Gerstel, Mühlheim, Germany). The 6 mL juice were mixed with 1.5 g NaCl in a 20-mL glass vial, and then 4 µL 2-octanol internal standard solution (53.84 mg/L) was added. The volatiles were extracted for 30 min using a SPME fiber coated with 50/30 μm divinylbenzene/carboxen/polydimethylsiloxane (DVB/CAR/PDMS; Supelco, Bellefonte, PA, USA) after 10 min of equilibration at 50 °C. The fiber was then immediately inserted into the gas chromatograph (GC) injection port for desorption at 260 °C for 3 min in splitless mode. Separation of the desorbed volatiles was carried out using an Agilent 7890 GC coupled with a 5975 mass-selective detector (Agilent Technologies, Santa Clara, CA, USA) equipped with a HP-Innowax column (30 m × 0.25 mm i.d., 0.25 µm film thickness; J & W Scientific, Folsom, CA, USA). The temperature program was as follows: 40 °C for 5 min, increased to 240 °C at 5 °C min^−1^, and then to 260 °C at 20 °C min^−1^ and held for 5 min. The flow rate of helium as carrier gas was 1 mL min^−1^. The mass spectrometry (MS) transfer line and ionization source temperature were 260 °C and 230 °C, respectively. Electron impact mass spectrometric data from *m/z* 20 to 400 were collected at 70 eV ionization voltages.

Compound identifications were performed via comparing mass spectral data with the National Institute of Standards and Technology (NIST) 2011 library and the retention times of the authenticated standards. For compounds that standards were unavailable in the library, tentative identifications were made via comparing their linear retention indices (LRI) and MS spectra with those reported in the literature. Compounds were quantified according to calibration curves, while the compounds without calibration curves were semi-quantified using the internal standard (Supplementary Table S3).

### Gene expression analyses

Total RNA was isolated from pulp using universal plant total RNA extraction kit (BioTeke, Beijing, China). RNA quality and concentration were assessed using agarose gel electrophoresis and a NanoDrop 2000 spectrophotometry (Thermo Scientific, Wilmington, DE, USA). cDNA was synthesized using Takara PrimeScript RT reagent Kit with gDNA Eraser (Takara, Dalian, China). The primer sequences used in present study are listed in Supplementary Table S4^8^. Quantitative real-time PCR (qRT-PCR) was conducted using a LightCycler 480 System (Roche, Mannheim, Germany) with SYBR Premix Ex Taq II (Takara, Dalian, China) as previously described^16^. Gene transcripts were quantified upon normalization to the internal reference genes *VvGAPDH* and *VvActin1*^45,46^ using geNorm software according to a previous report^47^.

### Statistical analysis

Data in this experiment were presented as the average of three replicates. One-way ANOVA (*p* < 0.05) and Duncan’s multiple range tests were conducted with SPSS 18.0 (SPSS Inc., IL, USA).

## Supplementary information

Supplementary Information.
