# Supplementary material for: Influence of cluster thinning and girdling on aroma composition in ‘Jumeigui’ table grape
Source: Sci Rep. 2020 Apr 23;10:6877. doi: 10.1038/s41598-020-63826-7 (PMC7181712; doi:10.1038/s41598-020-63826-7)
Supplement: Supplementary file 1 — Supplementary Information. [file 41598_2020_63826_MOESM1_ESM.docx]

**Influence of cluster thinningand girdling on aroma composition in ‘Jumeigui’ table grape**

Xiaojun Xi^1,2,*^, Qian Zha^1,2^, Yani He^1,2^, Yihua Tian^1,2^ and Aili Jiang^1,2,*^

^1^Forestry and Pomology Research Institute, Shanghai Academy of Agricultural Sciences, Shanghai 201403, China

^2^Shanghai Key Lab of Protected Horticultural Technology, Shanghai Academy of Agricultural Sciences, Shanghai 201403, China

^*^Corresponding author: Xiaojun Xi (email: xxj220401@126.com) or Aili Jiang (email: putaojal@163.com)


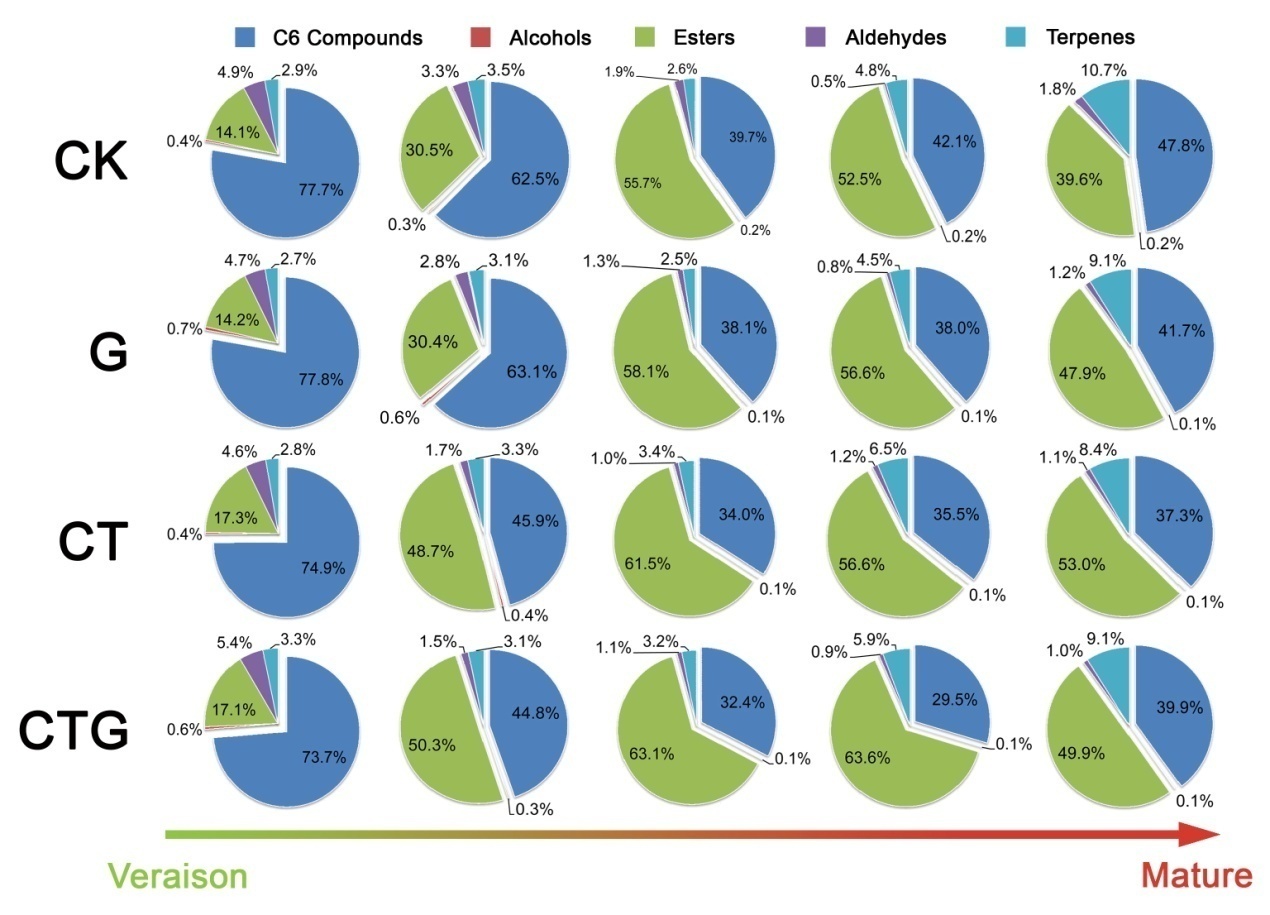


Supplementary Figure S1. Changes in ratio of aromatic compounds in response to cluster thinning and girdling (alone or in combination) of ‘Jumeigui’ grape from véraison to harvest. CK, the control treatment (unthinned and ungirdled); G, trunk girdled one week before véraison; CT, 50% clusters thinned one week before véraison; CTG, 50% clusters thinned and trunk girdled one week before véraison.


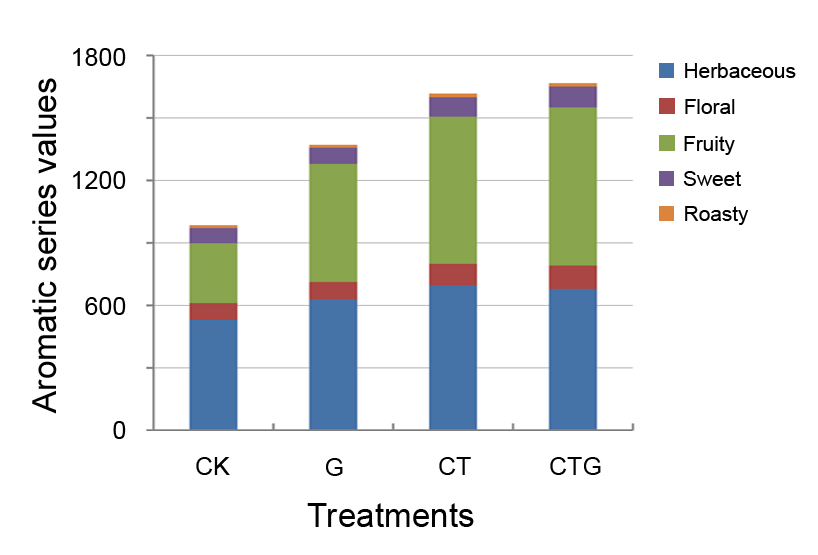


Supplementary Figure S2. Changes in aromatic series values in response to cluster thinning and girdling (alone or in combination) of ‘Jumeigui’ grape at harvest. CK, the control treatment (unthinned and ungirdled); G, trunk girdled one week before véraison; CT, 50% clusters thinned one week before véraison; CTG, 50% clusters thinned and trunk girdled one week before véraison.


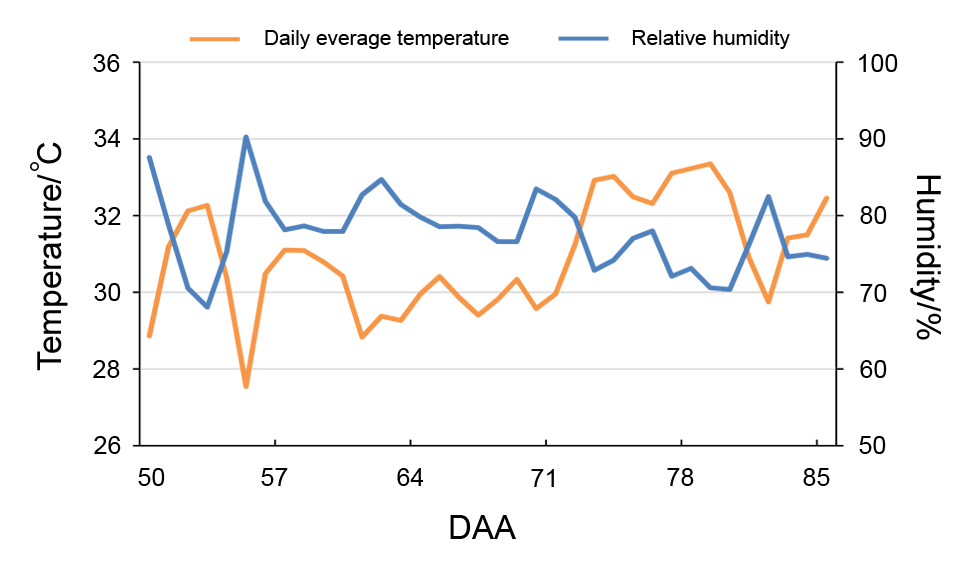


Supplementary Figure S3. Air temperature and relative humidity within the greenhouse throughout the experiment.

| Compounds | Sensory threshold  (μg/L) | Odorant series | Treatment | | | |
| --- | --- | --- | --- | --- | --- | --- |
|  |  |  | CK | G | CT | CTG |
| **C_6_ compounds** |  | |  | |  |  |
| Hexanal | 4.5^1^ | 1 | 368 | 413 | 455 | 463 |
| (*Z*)-3-Hexenal | 0.25^2^ | 1 | 32 | 35 | 44 | 53 |
| (*E*)-2-Hexenal | 17^3^ | 1 | 40 | 48 | 64 | 71 |
| Hexanol | 500^1^ | 1,2 | 0.9 | 0.8 | 1.3 | 1.7 |
| (*Z*)-3-Hexen-1-ol | 70^1^ | 1,7 | 0.2 | 0.2 | 0.2 | 0.2 |
| (*E*)-2-Hexen-1-ol | 100^4^ | 1 | 1.3 | 2.2 | 1.9 | 2.3 |
| **Alcohols** |  | |  | |  |  |
| 1-Octen-3-ol | 1^5^ | 8 | 3.3 | 3.1 | 4.8 | 7.3 |
| Heptanol | 425^1^ | 7 | <0.1 | <0.1 | <0.1 | <0.1 |
| 2-Ethyl hexanol* | 270^6^ | 2 | <0.1 | <0.1 | <0.1 | <0.1 |
| Octanol | 110^7^ | 2 | <0.1 | <0.1 | <0.1 | <0.1 |
| Phenylethyl alcohol | 1100^8^ | 2 | <0.1 | <0.1 | <0.1 | <0.1 |
| **Esters** |  | |  | |  |  |
| Ethyl acetate | 5000^1^ | 3,5,7,9,10 | 0.5 | 0.7 | 1.0 | 1.0 |
| Ethyl propionate* | 10^1^ | 3 | 0.4 | 0.5 | 0.5 | 0.9 |
| Ethyl isobutyrate | 15^9^ | 3 | nd | 1.0 | 1.8 | 3.7 |
| Ethyl butyrate | 1^1^ | 3 | 79 | 256 | 318 | 376 |
| Ethyl 2-methylbutanoate* | 0.091^4^ | 3 | 25 | 98 | 134 | 143 |
| Ethyl pentanoate | 1.5^2^ | 1 | 21 | 74 | 51 | 23 |
| Ethyl hexanoate | 1^1^ | 3 | 30 | 63 | 68 | 57 |
| Hexyl acetate | 670^10^ | 1,2,3 | <0.1 | <0.1 | <0.1 | <0.1 |
| 2-Hexenoic acidethyl ester* | 750^11^ | 1,3 | <0.1 | <0.1 | <0.1 | <0.1 |
| Ethyl octanoate* | 194^1^ | 2,3,4 | <0.1 | <0.1 | <0.1 | <0.1 |
| Ethyl 3-hydroxybutyrate* | 20000^12^ | 3,4,6 | <0.1 | <0.1 | <0.1 | <0.1 |
| **Aldehydes** |  | |  | |  |  |
| Pentanal | 12^1,7^ | 1,7 | 0.4 | 0.4 | 0.5 | 0.5 |
| Heptanal* | 3^13^ | 3,7 | 4.2 | 4.3 | 4.5 | 4.5 |
| Octanal | 0.7^1,2,7^ | 1,2,3,7 | 9.7 | 9.7 | 12.7 | 11.6 |
| Nonanal | 1^1,7^ | 1,3 | 60 | 48 | 65 | 54 |
| Benzaldehyde | 350^1^ | 2,3,4,6 | <0.1 | <0.1 | <0.1 | <0.1 |
| Phenylacetaldehyde* | 4^1^ | 2 | 1.2 | 0.7 | 0.8 | 0.9 |
| **Terpenes** |  | |  | |  |  |
| β-Myrcene | 36^8^ | 1,6 | 0.6 | 0.7 | 1.4 | 1.4 |
| D-Limonene | 10^8^ | 3 | 7.1 | 8.0 | 9.2 | 9.7 |
| β-cis-Ocimene* | 34^14^ | 1,3 | 0.4 | 0.4 | 0.5 | 05 |
| p-Cymene | 11.4^1^ | 1,3 | 0.2 | 0.2 | 0.3 | 0.3 |
| cis-Linalool oxide* | 0.5^8^ | 2 | 3.5 | 2.9 | 5.5 | 5.9 |
| Linalool | 6^8^ | 2,3,4 | 73 | 78 | 94 | 100 |
| α-Terpineol | 330^8^ | 2,4 | <0.1 | <0.1 | <0.1 | <0.1 |
| Nerol | 300^8^ | 1,2 | <0.1 | <0.1 | <0.1 | <0.1 |
| Geraniol | 40^8^ | 2 | 2.0 | 2.4 | 3.4 | 4.7 |

Supplementary Table S1. Odor activity values (OVAs) for volatile compounds in the pulp juice of ‘Jumeigui’ grape with cluster thinning and girdling at harvest. CK, the control treatment (unthinned and ungirdled); G, trunk girdled one week before véraison; CT, 50% clusters thinned one week before véraison; CTG, 50% clusters thinned and trunk girdled one week before véraison. Odour thresholds of the compounds were determined in water solution, except for ethyl isobutyrate, hexyl acetate and ethyl 3-hydroxybutyrate determined in ethanol-water solution. nd: not detected. * indicated the semi-quantitative determinations with the internal standards. Odorant series: 1, herbaceous; 2, floral; 3, fruity; 4, sweet; 5, spicy; 6, roasty; 7, fatty; 8, earthy; 9, balsamic; 10, solvent.

| Compound  Gene | Total terpenes | | | | Linalool | | | |
| --- | --- | --- | --- | --- | --- | --- | --- | --- |
|  | CK | G | CT | CTG | CK | G | CT | CTG |
| VvDXS1 | _ | _ | _ | _ | _ | _ | _ | _ |
| VvDXS3 | 0.970^**^ | 0.969^**^ | 0.973^**^ | 0.910^*^ | 0.940^*^ | 0.944^*^ | 0.942^*^ | 0.891^*^ |
| VvGPPS | 0.866 | 0.520 | 0.449 | 0.112 | 0.856 | 0.435 | 0.354 | 0.024 |
| VvCSLinNer | _ | _ | _ | _ | _ | _ | _ | _ |

Supplementary Table S2. The Pearson’s correlation coefﬁcients between terperne concentrations and gene expression levels in ‘Jumeigui’ grape berry. CK, the control treatment (unthinned and ungirdled); G, trunk girdled one week before véraison; CT, 50% clusters thinned one week before véraison; CTG, 50% clusters thinned and trunk girdled one week before véraison. * and** indicate significance at *p*<0.05 and *p* <0.01 (2-tailed), respectively; – indicate correlation coefﬁcient < 0.

| Compounds | Calibration graphs | r^2^ |
| --- | --- | --- |
| **C_6_ compounds** |  |  |
| Hexanal | y = 4.5912x - 0.1010 | 0.9975 |
| (*Z*)-3-Hexenal | y = 1.8035x - 0.0040 | 0.9992 |
| (*E*)-2-Hexenal | y = 4.5531x - 0.0035 | 0.9994 |
| Hexanol | y = 9.2890x - 0.1199 | 0.9965 |
| (*Z*)-3-Hexen-1-ol | y = 11.0240x - 0.0027 | 0.9853 |
| (*E*)-2-Hexen-1-ol | y = 13.9480x - 0.0047 | 0.9994 |
| **Alcohols** |  |  |
| 1-Octen-3-ol | y = 1.3250x - 0.0069 | 0.9980 |
| Heptanol | y = 1.1881x - 0.0076 | 0.9970 |
| Octanol | y = 0.9754x - 0.0027 | 0.9928 |
| Phenylethyl alcohol | y = 0.6585x - 0.0074 | 0.9912 |
| **Esters** |  |  |
| Ethyl acetate | y = 33.1230x - 0.1999 | 0.9981 |
| Ethyl isobutyrate | y = 6.4466x - 0.0788 | 0.9910 |
| Ethyl butyrate | y = 3.4627x - 0.0602 | 0.9818 |
| Ethyl pentanoate | y = 12.8954x + 0.0218 | 0.9980 |
| Ethyl hexanoate | y = 0.9127x + 0.0215 | 0.9983 |
| Hexyl acetate | y = 0.9114x - 0.0003 | 0.9990 |
| **Aldehydes** |  |  |
| Pentanal | y = 1.8075x - 0.0040 | 0.9967 |
| Octanal | y = 1.4606x - 0.0019 | 0.9965 |
| Nonanal | y = 3.9123x - 0.0087 | 0.9950 |
| Benzaldehyde | y = 7.3396x - 0.0116 | 0.9988 |
| **Terpenes** |  |  |
| β-Myrcene | y = 1.4759x + 0.0083 | 0.9989 |
| D-Limonene | y = 1.6730x - 0.0056 | 0.9994 |
| p-Cymene | y = 0.8372x - 0.0021 | 0.9999 |
| Linalool | y = 1.0045x - 0.0052 | 0.9995 |
| α-Terpineol | y = 2.2722x + 0.0023 | 0.9978 |
| Nerol | y = 2.6263x - 0.0117 | 0.9927 |
| Geraniol | y = 4.4518x + 0.0223 | 0.9961 |

Supplementary Table S3. Quantitative standards and calibration graphs for quantification of volatile compounds in ‘Jumeigui’ grapes. y, concentration ratio of a compound to the 2-octanol (internal standard); x, peak ratio of a compound to the 2-octanol; r, regression coefficient.

| Gene name | Accession number | Forward (5’→3’) | Reverse (5’→3’) |
| --- | --- | --- | --- |
| VvDXS1 | CU459219 | CTCATTTCCTGCCCATTTTAGC | CTTACTCCTTTGCTGGGATTGG |
| VvDXS3 | CU459223 | GAAGGCTCTGTTGGAGGGTTT | TCCTCTGGTGATGCCTGTTCT |
| VvDXR | CU459229 | AGAGGCTTTGGCTGACTGTGA | AACCTGCGCAACCTACTATTCC |
| VvHDR | CU459225 | TCTTCCTCGTCTGTGGCTGTT | GCGATTCATGAGCTCCAGAGT |
| VvGPPS | AY351862 | AGAATCTGGGATTGGCATTCC | TGGCGGATGTCAGACAATGA |
| VvCSLinNer | HM807393 | TGGGATTCTCTCCTGCCTTTT | GCAGTAGGCACAAGCACAACA |
| VvActin1 | XM_002282480 | CTTGCATCCCTCAGCACCTT | TCCTGTGGACAATGGATGGA |
| VvGAPDH | VIT_17s0000g10430 | TTCCGTGTTCCTACTGTTG | CCTCTGACTCCTCCTTGAT |

Supplementary Table S4. Primers used for the quantification of gene expression levels by qRT-PCR of ‘Jumeigui’ grape.

**Supplemental References list**

1. Pino, J. A. & Mesa, J. Contribution of volatile compounds to mango (*Mangifera indica* L.) aroma. *Flavour Fragrance J* **21**, 207–213, doi:10.1002/ffj.1703 (2006).

2. Genovese, A., Dimaggio, R., Lisanti, M. T., Piombino, P. & Moio, L. Aroma composition of red wines by different extraction methods and Gas Chromatography-SIM/MASS spectrometry analysis. *Ann Chim* **95**, 383–394, doi:10.1002/adic.200590045 (2005).

3. Genovese, A., Lamorte, S. A., Gambuti, A. & Moio, L. Aroma of Aglianico and Uva di Troia grapes by aromatic series. *Food Res Int* **53**, 15–23, doi:10.1016/j.foodres.2013.03.051 (2013).

4. Qian, M. C. & Wang, Y. Seasonal variation of volatile composition and odor activity value of ‘Marion’ (*Rubus spp*. hyb) and ‘Thornless Evergreen’ (*R. laciniatus* L.) blackberries. *J Food Sci* **70**, 13–20, doi:10.1111/j.1365-2621.2005.tb09013.x (2005).

5. Yang, C. *et al*. Common aroma-active components of propolis from 23 regions of China. *J Sci Food Agric* **90**, 1268–1282, doi:10.1002/jsfa.3969 (2010).

6. Pino, J. A. & Queris, O. Analysis of volatile compounds of mango wine. *Food Chem* **125**, 1141–1146, doi:10.1016/j.foodchem.2010.09.056 (2011).

7. Buttery, R. G., Turnbaugh, J. G. & Ling, L. C. Contribution of volatiles to rice aroma. *J Agric Food Chem* **36**, 1006–1009, doi:10.1021/jf00083a025 (1988).

8. Fenoll, J., Manso, A., Hellin, P., Ruiz, L. & Flores, P. Changes in the aromatic composition of the *Vitis vinifera* grape Muscat Hamburg during ripening. *Food Chem* **114**, 420–428, doi:10.1016/j.foodchem.2008.09.060 (2009).

9. Guth, H. Identification of character impact odorants of different white wine varieties. *J Agric Food Chem* **45**, 3022–3026, doi:10.1021/jf9608433 (1997).

10. Franco, M., Peinado, R. A., Medina, M. & Moreno, J. Off-vine grape drying effect on volatile compounds and aromatic series in must from Pedro Ximénez grape variety. *J Agric Food Chem* **52**, 3905–3910, doi: 10.1021/jf0354949 (2004).

11. Aparicio, R. & Morales, M. T. Characterization of Olive Ripeness by Green Aroma Compounds of Virgin Olive Oil. *J Agric Food Chem* **46**, 1116–1122, doi:10.1021/jf970540o (1998).

12. González álvarez, M., González-Barreiro, C., Cancho-Grande, B. & Simal-Gándara, J. Relationships between Godello white wine sensory properties and its aromatic fingerprinting obtained by GC–MS. *Food Chem* **129**, 890–898, doi:10.1016/j.foodchem.2011.05.040 (2011).

13. Wang, L., Baldwin, E. A., Plotto, A., Luo, W., Raithore, S., Yu, Z. & Bai, J. Effect of methyl salicylate and methyl jasmonate pre-treatment on the volatile profile in tomato fruit subjected to chilling temperature. *Postharvest Biol Tec* **108**, 28–38, doi:10.1016/j.postharvbio.2015.05.005 (2015).

14. Du, X., Finn, C. E. & Qian, M. C. Volatile composition and odour-activity value of thornless ‘Black Diamond’ and ‘Marion’ blackberries. *Food Chem* **119**, 1127–1134, doi:10.1016/j.foodchem.2009.08.024 (2010).
